# Anthocyanin (ATH)-incorporating polyvinylpyrrolidone-ethyl cellulose-(2-hydroxypropyl)-β-cyclodextrin (PVP–EC–BCD) nanofiber-based pH sensor for ocular pH detection during accidental chemical spills

**DOI:** 10.1039/d5na00819k

**Published:** 2025-11-26

**Authors:** Benuwan Sandaruwan, Renuka Liyanage, Pabakara Costha, Rohan S. Dassanayake, Ruchire Eranga Wijesinghe, H. M. L. P. B. Herath, K. M. Nalin de Silva, Rohini M. de Silva, Suranga M. Rajapaksha, Udaya Wijenayake, Danushika C. Manatunga

**Affiliations:** a Department of Biosystems Technology, Faculty of Technology, University of Sri Jayewardenepura Pitipana North, Homagama 10200 Sri Lanka danushi@sjp.ac.lk; b Department of Materials and Mechanical Technology, Faculty of Technology, University of Sri Jayewardenepura Pitipana North, Homagama 10200 Sri Lanka; c National Eye Hospital of Sri Lanka Colombo 01000 Sri Lanka; d Center for Excellence in Informatics, Electronics & Transmission (CIET), Sri Lanka Institute of Information Technology Malabe 10115 Sri Lanka; e Department of Electrical and Electronic Engineering, Faculty of Engineering, Sri Lanka Institute of Information Technology Malabe 10115 Sri Lanka; f Center for Advanced Materials and Devices (CAMD), Department of Chemistry, University of Colombo PO Box 1490 Colombo 00300 Sri Lanka; g Department of Life Sciences, Faculty of Science, NSBM Green University Homagama 10200 Sri Lanka; h Department of Computer Engineering, Faculty of Engineering, University of Sri Jayewardenepura Nugegoda 10250 Sri Lanka

## Abstract

The existing ocular pH detection methods encounter numerous limitations, including low accuracy, poor sensitivity across a wide pH range, and patient discomfort, highlighting the need for innovative approaches. A novel biosensor for ocular pH detection has been developed to assess ocular health and chemical injuries in clinical settings. This study uses the pH-sensitive properties of anthocyanins (ATHs), natural pigments extracted from butterfly pea flowers, to develop a novel pH-responsive nanofiber mat. ATHs are integrated into a polymer blend containing polyvinylpyrrolidone (PVP), ethyl cellulose (EC), and (2-hydroxypropyl)-β-cyclodextrin (BCD) to fabricate electrospun nanofibers. The acquired characterization, employing scanning electron microscopy (SEM), Fourier-transform infrared spectroscopy (FTIR), X-ray diffraction (XRD), and thermogravimetric analysis (TGA), confirmed the successful fabrication of the ATH-infused nanofibers with a mean diameter ranging from 121 to 396 nm. Four formulations were tested: PVP:EC:BCD:ATH (18 ppm), PVP:EC:BCD:ATH (25 ppm), PVP:EC:BCD:ATH (35 ppm), and PVP:EC:BCD:ATH (50 ppm). Among them, the 50 ppm ATH-incorporating nanofiber mat exhibited the best performance in terms of color clarity, response time, and pH sensitivity. The fabricated 50 ppm ATH incorporating nanofiber mat demonstrated a rapid pH response time of less than 5 seconds (s) while exhibiting a color variation from pink to blue to green across the pH range of 1 to 12, providing a rapid and accurate method for visual pH detection. Based on the color performance of the 50 ppm ATH-incorporating system, a standardized color reference chart was developed to serve as a practical and visual guide for estimating pH levels in clinical applications. Zebrafish toxicity assays were conducted further to validate the safety and biocompatibility of the developed sensor, revealing no significant toxic effects across the range of ATH concentrations.

## Abstract 2

The study presents a novel ATH-incorporated PVP–EC–BCD electrospun nanofiber mat as a rapid, biocompatible ocular pH sensor. It overcomes key limitations of current methods, such as low accuracy, limited sensitivity across wide pH ranges, and patient discomfort.

## Graphical abstract

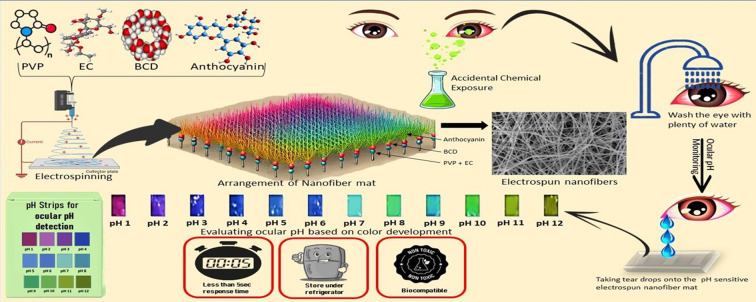

## 1 Introduction

Monitoring the pH level of the eye is crucial in point-of-care settings in ophthalmic clinics due to its broad implications. Generally, the monitoring process includes assessing and treating ocular chemical spillages and microbial infections, evaluating tear buffering capacity, and estimating the penetration of ocular drugs.^1–4^ The normal pH of the eye is around pH 7.1, which may vary depending on the ocular condition, such as rosacea, ocular infections, or chemical spills.^5–7^ Chemical spills can cause sudden alterations in ocular pH compared to other conditions. Among ocular traumas, approximately 20% are caused by chemical burns.^8,9^ These burns give rise to severe damage to the eye, with alkali chemicals being more detrimental than acidic chemicals.^10^ The severity of ocular injury depends on several factors, such as the type of chemical, volume, pH, and duration of exposure. However, the pH or concentration of the chemical is the most critical parameter related to the severity of ocular chemical burns.^7^ Therefore, immediate evaluation and management of ocular chemical burns are essential to prevent possible damage to the eye.^11^ Precise measurement of ocular pH is crucial in managing chemical burns in the eye, as it helps determine the most effective treatment approach.^8^ During an ocular chemical burn, the first step is to thoroughly wash the eye with plenty of water or use pH-neutralization solutions that remove the exposed chemical(s) and bring the ocular pH back to a neutral level.^12^ The initial ocular pH measurement determines the amount of water or neutralization solution required for irrigation to return the ocular pH to the normal range.^13^ However, the failure to correctly assess the ocular pH after excessive washings results in trace amounts of alkali or acid residues retained in the conjunctiva or on the ocular surface. These residual chemicals may penetrate deeper into the eye over time, leading to more severe complications, including corneal scarring, stromal necrosis, or even complete vision loss.^14^ Alkali substances are especially known for causing more severe tissue damage, penetrating the eye more quickly and deeply compared to acidic substances. Inaccurate pH measurements in such cases might delay appropriate treatment and further progression of injury that could have otherwise been prevented. This underscores the importance of detecting accurate pH promptly, which is necessary for appropriate therapeutic interventions.^15^ Once the ocular pH returns to the normal range, treatments can be implemented immediately.^16^ In emergency departments and ophthalmic clinics, the pH of human tears or ocular surfaces is measured using various methods, including litmus papers,^17^ bromothymol (BTB) dye,^17^ nitrazine strips,^17^ glass electrodes,^18^ fluorescent probes,^19^ glass probes,^20^ contact lenses,^21^ and microelectrodes.^3,21^ However, many of these ocular pH detection methods encounter several challenges. For instance, nitrazine strips are unsuitable for alkaline chemical spills with pH values above 7.5, while bromothymol blue (BTB) is limited to a narrow pH range of 6.0 to 7.6. Litmus papers exhibit low accuracy, and the methods involving glass probes and contact lenses cause patient discomfort. These limitations highlight the need for a simple, user-friendly approach to ocular pH detection during chemical spills.^8,16,22^

ATHs are water-soluble and highly pH-sensitive dye molecules that exhibit distinct color changes in response to pH variations.^23–25^ ATHs are natural pigments in many vascular plants and are glycosylated polyhydroxy and polymethoxy derivatives of 2-phenylbenzopyrylium, such as the flavylium cation.^26^ ATHs perform various functions in plants, including protection, propagation, repelling parasites, safeguarding plants against biotic and abiotic stress, and attracting pollinators and seed dispersers.^27^ In healthcare, ATHs offer various health benefits, such as anti-diabetic, neuroprotective, antioxidant, anti-carcinogenic, prevention of cardiovascular disease, and anti-inflammatory properties.^27,28^ In ocular healthcare, ATHs have been used to manage various inflammatory diseases and circulatory disorders,^29,30^ including retinal inflammation^31^ and diabetic retinopathy.^32^ ATHs can be extracted from various natural sources, including grapes,^33^ berries,^34^ red cabbage,^35^ radishes,^36^ and butterfly pea flowers (*Clitoria ternatea* L.).^37^ Recent studies have explored ATH-incorporating materials for pH sensing in fields like food packaging,^38^ textile dying,^39^ and cosmetics.^40^

ATH-infused pH sensors generally consist of a supportive matrix and the ATH dye.^41^ A notable example is the development of biodegradable, pH-responsive films using ATHs extracted from banana bracts for intelligent food packaging, as reported by Thottathil Nazar *et al.* (2024).^42^ These films, fabricated by incorporating ATHs into a polyvinyl alcohol (PVA)/starch matrix through the solution casting method, exhibited distinct color changes in response to pH variations. The pH responsiveness of the films was evident through measurable shifts in lightness, redness, yellowness, and blueness values, allowing for the visual detection of changes in environmental pH.^42^ The work conducted by Prietto *et al.* developed a pH indicator by electrospinning purple cabbage ATH extract with zein polymer, proposing it for the active food packaging industry. This pH sensor was capable of making color changes within the pH range of 1 to 10, which the human eye can identify.^43^ Lv *et al.* developed a starch-ATH-based pH-sensitive electrospun nanofiber mat for real-time food freshness monitoring. The results revealed color visibility and a reversible pH response, where roselle (*Hibiscus sabdariffa*) was employed to extract the ATH pigment.^44^ Liu *et al.* developed a colorimetric nanofiber sensor to evaluate shrimp freshness utilizing polycaprolactone (PCL) polymer with *Clitoria ternatea* Linn ATH.^45^ Interestingly, the pH-responsive properties of ATHs can be employed to develop pH sensors suitable for ocular pH detection.^28,46^ However, the use of ATHs in ocular pH detection applications is scarce.^21^

Electrospinning is an emerging technique widely utilized for fabricating ATH-based pH sensors, primarily in food packaging, textile materials development, and environmental monitoring applications.^28,41–48^ In recent years, electrospinning and electrospraying have advanced dramatically, providing researchers with new tools for tailoring nanofiber architectures and functionalities. The evolution of electrospinning from single-fluid blending to complex multi-fluid systems such as coaxial, side by side, triaxial, and Janus configurations has expanded the fabrication of functional nanostructures with distinct physicochemical properties. For instance, Deng Guang Yu *et al.* (2025) highlighted the unique potential of electrospun Janus nanofibers that enable dual drug loading and multifunctional wound dressing performance.^49^ Shu Chen *et al.* (2025) demonstrated a one-step side-by-side electrospraying approach for creating bioinspired Janus particles with multifunctional properties such as antibacterial activity and UV resistance.^50^ Similarly, Yaoning Chen *et al.* (2025) reported coaxial electrospinning as an efficient route for designing core–shell nanofibers with controlled drug release, while Yujie Hu *et al.* (2025) developed a tri-layer core sheath nanofibrous coating for sequential therapeutic delivery in orthopedic implants.^51,52^ Weiqiang Wang *et al.* (2025) emphasized the growing importance of green electrospinning techniques to replace toxic solvents in large-scale manufacturing, while Pu Wang *et al.* (2025) used coaxial electrospinning to design ZIF-8@PAN nanofibers for efficient environmental remediation.^53,54^ Collectively, these studies demonstrate how innovations in electrospinning and electrospraying are expanding the versatility of nanofibers across biomedical, environmental, and sensing applications. Incorporating this context, the current study aligns with the ongoing evolution of electrospinning science, leveraging its potential to produce stable and biocompatible nanofiber matrices for ocular pH detection.

Despite its extensive use in detecting pH variations in these fields, the application of ATH-based pH sensors in biomedical and clinical settings remains limited, with only a few studies exploring their potential.^21,55,56^ The use of electrospinning techniques to develop matrices for ATH-based pH sensors provides high surface area, high porosity, small pore size, and high absorption capacity, making them highly suitable for biomedical applications.^43,55,57–59^ In this regard, electrospun nanofiber matrices have demonstrated promising capabilities in wound healing applications, where their biocompatibility and pH-responsive properties facilitate monitoring of infection-related pH fluctuations. However, their potential in ocular diagnostics remains largely unexplored. Electrospinning is widely used for preparing non-woven fiber mats or membrane matrices using polymer solutions.^60–62^ Electrospun nanofiber matrices possess a high surface-area-to-volume ratio, allowing for rapid interaction with the teardrop or ocular surface, while the porosity ensures efficient absorption and retention of the ATH dye.^63,64^ Additionally, the small pore size provides a controlled environment for the dye, improving its stability and responsiveness to pH changes. These features collectively make nanofiber mats an ideal candidate for real-time ocular pH monitoring, offering significant advantages over traditional materials, such as pH paper strips or contact lenses.

PVP is one of the commonly used polymers for electrospinning, which is a biocompatible polymer approved by the United Nations Food and Drug Administration (US FDA). Its rapid dissolution capability also makes it a preferred ingredient in various drug formulations.^65,66^ EC and BCD polymers are also employed in electrospinning due to their unique features. EC offers excellent biocompatibility and hydrophobic properties, while BCD is also a biocompatible polymer with high loading capacity.^65,67,68^

The current study describes the preparation and characterization of an ATH-incorporating polyvinylpyrrolidone-ethyl cellulose-(2-hydroxypropyl)-β-cyclodextrin (PVP–EC–BCD) nanofiber-based pH sensor for detecting ocular pH during a chemical spill. Our work represents a significant advancement by integrating ATHs into an electrospun nanofiber matrix mat prepared from a novel formulation of PVP, EC, and BCD. The ATH-incorporating PVP–EC–BCD nanofiber-based pH sensor was characterized using SEM, FTIR, XRD, and TGA. Zebrafish acute toxicity studies were also performed to investigate biocompatibility and cytotoxicity. The validation of the pH sensor was conducted using color variation analysis using originally captured images without prior post-processing. The shelf-life assessment of the ATH-incorporating nanofiber mat was investigated under different storage conditions to determine its stability. To the best of our knowledge, this is the first attempt to report an ATH-incorporating PVP–EC–BCD nanofiber-based pH sensor for ocular pH detection, addressing the limitations of existing ocular pH detection methods and providing a more effective solution for ophthalmic clinics.

## 2 Materials and methods

### 2.1 Materials

Butterfly pea flowers (*Clitoria ternatea*) were collected from a local supplier in Sri Lanka. PVP (average molecular weight 360 000 Da), EC (48% ethoxy, 4 cP), BCD (average molecular weight 1460 Da), and absolute ethanol (≥99.8%) were purchased from Sigma-Aldrich (Saint Louis, MO). All other chemicals used were of analytical grade and purchased from Sigma-Aldrich (Saint Louis, MO), and water was doubly distilled before use.

### 2.2 Methods

#### 2.2.1. ATH extraction from butterfly pea (*Clitoria ternatea*) flowers

ATH extraction was conducted using a method described elsewhere.^60^ Initially, 25.0 g of the butterfly pea flowers was weighed and put into 25 mL of acidified absolute ethanol solution (5.67 (absolute ethanol) : 1 (HCl, 1.5 M) v/v). The mixture was subjected to constant agitation for 1 h at room temperature (RT). After the agitation, the supernatant was separated. Then, an additional 25 mL of acidified absolute ethanol was added to the sample, and the mixture was subjected to constant agitation for 1 h at RT. After that, the supernatant was again separated. Then, the separated supernatants were centrifuged using a centrifuge machine (R-300 Buchi, Flawil, Switzerland) for 15 min at 3500 rpm at RT. The resulting secondary supernatant was then separated and stored at 4 °C under dark conditions until further use.

#### 2.2.2. ATH content calculation

The ATH content was calculated using the pH differential method reported previously.^61^ The value obtained here denotes the equivalents of cyanidin 3-glucoside (Cy 3-glc). MW represents the molecular weight of Cy 3-glc (449.2 g mol^−1^), DF represents the dilution factor, and *ε*_m_ represents the molar extinction coefficient of Cy 3-glc, which is 26 900.^69^ The content of the ATH was determined using eqn (1).1
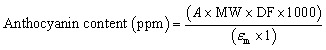


The absorbance (*A*) was measured using a Thermo Scientific Genesys 20 UV-vis spectrophotometer at two pH values (1.0 and 4.5), for which the solutions were prepared using a potassium chloride–hydrochloric acid buffer and sodium acetate–acetic acid buffer, respectively. The absorbance was recorded at the maximum wavelength corresponding to the ATH pigment. The differential absorbance values obtained at the two pH levels were used in the above equation (eqn (1)) to calculate the ATH content. Eqn (2) represents the value for *A* within the visible range.

2 *A* = (*A* − *A*_700_) _pH 1.0_ − (*A*_30_ − *A*_700_) _pH 4.5_

#### 2.2.3. Preparation of electrospinning solution

PVP, EC, BCD, and ATH were dissolved in 80% (v/v) ethanol aqueous solution under magnetic stirring for 12 h at RT to obtain a homogeneous solution. The total polymer concentration of PVP and EC was maintained at 10% w/v of the total volume of the electrospinning solution, where the w/w component ratio of PVP to EC was 3 : 1. To prepare the electrospinning solution, 0.225 g of PVP and 0.075 g of EC were added to 3 mL of 80% ethanol. The amount of BCD was also maintained constant, and only the ATH content varied during the sample preparation. Table 1 shows the amounts of PVP, EC, BCD, and ATH used to prepare electrospinning solutions.

**Table 1:** The amounts of PVP, EC, BCD, and ATH used in electrospinning

PVP:EC ratio (w/w)	BCD amount (g)	ATH concentration (ppm)	Total volume (mL)
3 : 1	—	—	3.0
3 : 1	0.025	—	3.0
3 : 1	0.025	18	3.0
3 : 1	0.025	25	3.0
3 : 1	0.025	35	3.0
3 : 1	0.025	50	3.0

#### 2.2.4. Electrospinning procedure

The prepared electrospinning solutions were loaded into a 5 mL plastic Terumo syringe fitted with a metal spinneret (20 G, internal diameter 0.61 mm). To dispense the electrospinning solution at a flow rate of 1.5 mL h^−1^, a syringe pump (KDS100, Cole-Parmer, USA) was used. The power supply (ZGF-2000, Shanghai Sute Electrical Co. Ltd, China) was employed to supply a high voltage of 22 kV. The distance between the needle and the grounded collector was kept at 16 cm, and the grounded collector was covered with aluminum foil. The electrospinning was conducted at a temperature of 18 °C for 2 h. The collected fiber mats were then stored in a vacuum desiccator at RT to remove residual moisture.

#### 2.2.5. Nanofiber characterization

##### 2.2.5.1. SEM

The morphological and surface parameters of electrospun fibers were examined using SEM images on a Carl Zeiss Evo 18 SEM (accel. voltage: 10 kV, probe current: 1–25 pA, resolution: 1024 px × 768 px). The images were analyzed using ImageJ software (National Institutes of Health).

##### 2.2.5.2. FTIR spectroscopy

FTIR spectra of the nanofiber mats were obtained using a PerkinElmer Spectrum 100 instrument in the wavelength region of 400–4000 cm^−1^ in the attenuated total reflectance (ATR) mode at a resolution of 4 cm^−1^. All spectra were recorded with 32 scans across the wavelength range of 400–4000 cm^−1^.

##### 2.2.5.3. XRD

Powder X-ray diffraction (XRD) patterns of the nanofibers were obtained using a Rigaku (SmartLab SE, Japan) powder X-ray diffractometer over the two theta (2*θ*) range of 5°–80° using Cu Kα radiation, with a step size of 2*θ* = 5° min^−1^, an accelerating voltage of 40 kV, and a tube current of 44 mA.

##### 2.2.5.4. TGA

TGA was performed on a high-resolution TGA Q-500 analyzer (TA Instruments, Inc., New Castle, DE). Samples with weights ranging from 5–10 mg were heated from 30 to 700 °C under a nitrogen (N_2_) atmosphere at a heating rate of 10 °C min^−1^ in high-resolution mode.

#### 2.2.6. Zebrafish embryo toxicity assay (FET 236 acute toxicity assessment)

Adult male and female zebrafish (*Danio rerio*) were selected and set up for breeding. They were kept in a specialized mini-zebrafish facility at the Biotechnology Laboratory of the University of Colombo, Sri Lanka, following established protocols.^70,71^ Embryos at 48 h post-fertilization (hpf) were gathered from overnight breeding sessions in a freshly prepared embryo medium. The embryos were sorted into Petri dishes, discarding any that were unfertilized, coagulated, damaged, opaque, asymmetric, or lacked transparency. They were then gently washed two or three times with distilled water while being carefully examined under a Meichoon Digital Microscope 3-in-1 USB Interface Camera, and the ‘Hi View’ application was used for the observations. Ten zebrafish embryos at 24 h post-fertilization (hpf) were then carefully placed in each 6 well plate containing 5 mL of AntiClo-treated water. Pieces of electrospun nanofiber mats, each having a size of 1 cm^2^, were placed into each well of a six well plate. The wells contained different mats: a neat PVP:EC:BCD polymer mat, an 18 ppm ATH-incorporating PVP:EC:BCD polymer mat, a 25 ppm ATH-incorporating PVP:EC:BCD polymer mat, a 35 ppm ATH-incorporating PVP:EC:BCD polymer mat, and a 50 ppm ATH-incorporating PVP:EC:BCD polymer mat. One well was left without adding any mat as a control.^72^ Each treatment was conducted in triplicate at around 28.5 °C. Zebrafish embryo hatching and mortality were evaluated and recorded at 24, 48, 72, and 96 hpf. Heart rate was documented at 72 hpf, while deformities were documented at 96 hpf.

#### 2.2.7. Color variation analysis

The polymeric nanofiber mats incorporating different ATH concentrations (18, 25, 35, and 50 ppm) were cut into 1 × 1 cm dimensions and then exposed to buffer solutions with pH values ranging from 1 to 12. Images of the mats at each concentration and pH level were captured using a Sony Cyber-shot DSC-W320 camera, positioned 16 cm away from the samples under controlled laboratory lighting at 200 lux to ensure consistency. Each image was cropped into a 50 px × 50 px section to maintain uniformity across all samples. These cropped images were imported into ImageJ software for histogram analysis, where the mean values of the red (R), green (G), and blue (B) color channels were calculated to quantify color variation.

These R, G, and B coordinates provided a measure of color variation for each sample across the different pH levels. The R, G, and B values were plotted as bar graphs for each ATH concentration to represent the color variations and identify significant trends.

#### 2.2.8. Shelf-life evaluation

The shelf-life evaluation of the pH-sensitive electrospun nanofiber mats was conducted to determine the optimal storage conditions for maintaining their intact behavior over time. The nanofiber mats were stored under light, dark, and refrigerated conditions for three months. The mats were tested every two weeks to evaluate their pH-responsive ability. For each evaluation, buffer solutions with selected pH values of 3, 5, 7, 9, and 11 were applied to the nanofiber mats to observe their color response. This process was repeated every two weeks for three months, allowing a comparative analysis of how storage conditions impact the stability and pH sensitivity of the nanofiber mats over time. The results from these tests were used to identify the optimal storage conditions for pH-sensitive electrospun nanofiber mats.

## 3 Results and discussion

### 3.1 Morphological analysis

**Fig. 1:**
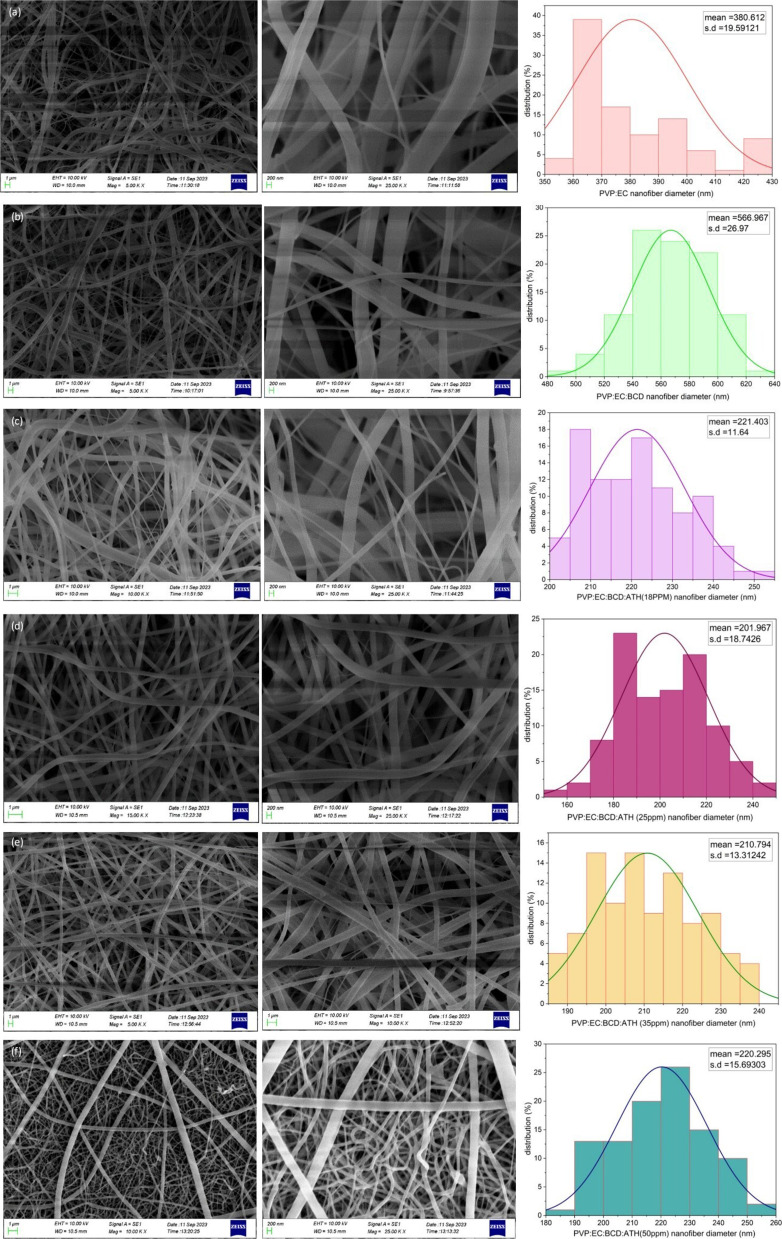
SEM images of different electrospun nanofiber mats and their mean diameters: (a) PVP:EC, (b) PVP:EC:BCD, (c) PVP:EC:BCD:18 ppm ATH. (d) PVP:EC:BCD:25 ppm ATH. (e) PVP:EC:BCD:35 ppm ATH. (f) PVP:EC:BCD:50 ppm ATH.

Fig. 1 depicts the SEM images of all electrospun nanofiber mats and their mean diameters. As can be seen from Fig. 1, all electrospun fibers exhibited smooth and cylindrical morphologies. The mean diameters of neat PVP:EC and PVP:EC:BCD systems were 380.61 ± 19.59 and 566.97 ± 26.97 nm, see Fig. 1(a) and (b). The diameter of the nanofibers increased after adding BCD. The mean diameters of PVP:EC:BCD:ATH (18 ppm), PVP:EC:BCD:ATH (25 ppm), PVP:EC:BCD:ATH (35 ppm), and PVP:EC:BCD:ATH (50 ppm) were 221.40 ± 11.64 nm, 201.97 ± 18.74 nm, 210.79 ± 13.31 nm, and 220.30 ± 15.69 nm, respectively, see Fig. 1(c)–(f). The variation in diameter among the ATH-containing samples is minimal, and a notable reduction in fiber diameter is observed when comparing the ATH-loaded nanofibers to the PVP:EC:BCD and PVP:EC formulations without ATH. This could be due to the change in the viscosity of the electrospinning solution after adding ATH.^73^ This viscosity reduction would make it easier to stretch the electrospinning jet and generate nanofibers with lower diameters.^74^ Additionally, ATH molecules could develop surface charges on the electrospinning jet, enhancing the elongation of the jet and reducing the fiber diameter by increasing electrostatic repulsion between similarly charged polymer chains and ATH molecules within the electrospinning jet.^75,76^

A visual difference in surface fiber thickness was observed among the samples, particularly between Fig. 1(a)–(c) and (f), although their mean diameters were different. This apparent variation arises from differences in fiber deposition density and spatial arrangement on the collector rather than an actual increase in individual fiber diameter. During electrospinning, small fluctuations in charge distribution and solvent evaporation rate can alter the jet trajectory and fiber packing orientation. This can lead to localized regions where fibers overlap or align sparsely or densely, thereby appearing as thicker or thinner fibers under a SEM. Such morphological heterogeneity is typical of multi-component electrospinning systems and does not represent any structural irregularity or abnormality.^77^

### 3.2 FTIR analysis

**Fig. 2:**
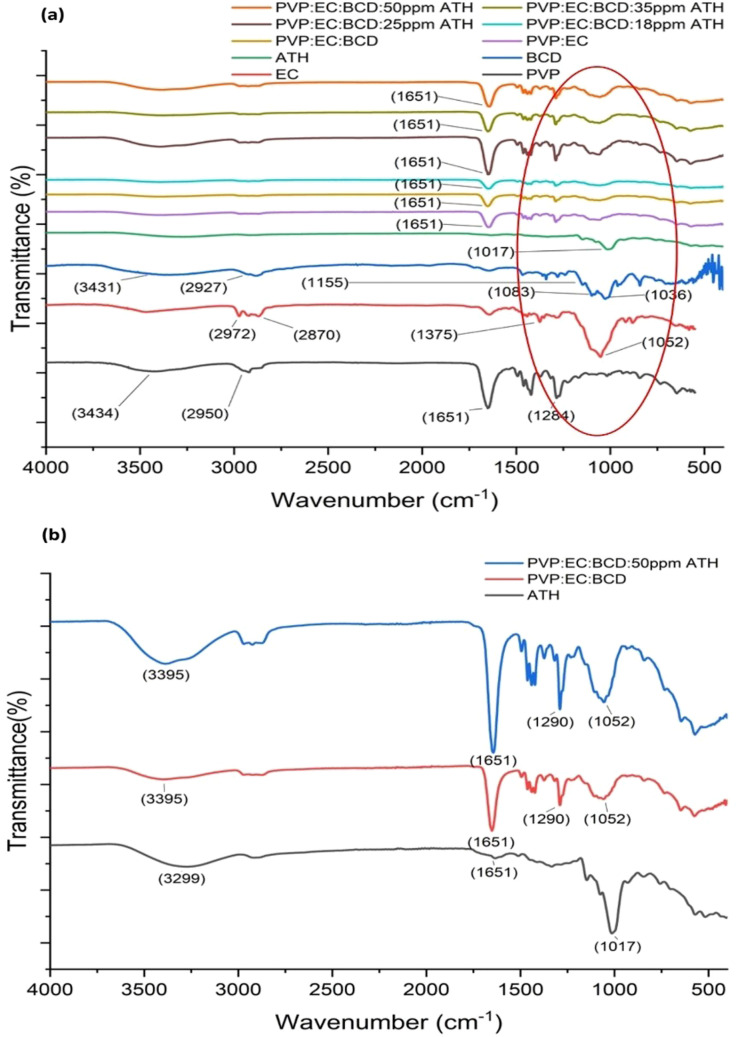
(a) FTIR spectra of neat polymers (PVP, EC, BCD), ATH and different ATH concentration incorporating electrospun nanofiber mats (PVP:EC:BCD:18 ppm ATH, PVP:EC:BCD:25 ppm ATH, PVP:EC:BCD:35 ppm ATH and PVP:EC:BCD:50 ppm ATH) in the 4000–400 cm^−1^ wavelength range and (b) enlarged FTIR spectra of neat ATH, neat PVP:EC:BCD nanofiber mat, and 50 ppm ATH incorporating PVP:EC:BCD nanofiber mat.

FTIR analysis was performed to investigate the chemical properties of the nanofiber mats prepared. The chemical structures of the compounds used are shown in Fig. S1 (SI). The different functional groups of the electrospun nanofibers and neat polymers could be identified by the FTIR spectra shown in Fig. 2. Fig. 2(a) shows the FTIR spectra of different ATH-incorporating electrospun nanofibers and neat polymers. In the PVP spectrum (see Fig. 2(a)), the characteristic peaks that appear at 1651 cm^−1^ corresponded to the C=O stretching vibrations, whereas the band at 1284 cm^−1^ is attributed to the C–N stretching.^65,78^ The characteristic bands at 2950 and 3434 cm^−1^ are ascribed to the –C–H symmetric and –O–H symmetric stretching vibrations of PVP.^79^ In the blank EC spectrum depicted in Fig. 2(a), the characteristic peaks at 1052 cm^−1^ and 1375 cm^−1^ correspond to the C–O–C stretching and –CH bending, respectively. Furthermore, the 2972 cm^−1^ and 2870 cm^−1^ bands indicated the –CH stretching vibrations of EC.^65^ The band at 3431 cm^−1^ of BCD is due to the –OH group vibration, while that at 2927 cm^−1^ is attributed to the –CH vibrations (Fig. 2(a)). The characteristic peaks at 1155 cm^−1^, 1083 cm^−1^, and 1036 cm^−1^ are ascribed to the vibrations of C–O, C–O–C, C–C–O, and C–C–C asymmetric valence vibrations of BCD.^80^ As shown in Fig. 2(b), the absorption peak at 1017 cm^−1^ represents the C–O(H) groups of ATH molecules.^81^ The peak at 1651 cm^−1^ is attributed to the C=O stretching vibration of ATH molecules.^82^ The broad band at 3299 cm^−1^ is ascribed to O–H stretching, confirming the presence of hydroxyl functional groups, which play a critical role in facilitating hydrogen bonding interactions with the polymer matrix.^83^

The characteristic peak at 1281 cm^−1^ represents the –CO group stretching of the PVP:EC polymer blend,^84^ and when BCD is incorporated into the PVP:EC polymer blend, the position of this peak is slightly shifted to 1290 cm^−1^, as shown in Fig. S2. After the incorporation of ATH, there is no change in the position of the peak at 1290 cm^−1^; nevertheless, an increase in intensity is observed (see Fig. 2(b)). This increased intensity suggests enhanced hydrogen bonding interactions between the ATH molecules and the polymer blend, potentially due to the presence of more hydroxyl groups from the ATH, which could lead to stronger intermolecular forces within the system.^85,86^ The observed increase in the intensity of peaks, particularly around 3395 cm^−1^ (O–H stretching) and 1651 cm^−1^ (C=O stretching), indicates stronger and more efficient interaction between ATH and the polymer matrix components.^87^

It is important to note that no new distinct absorption peaks characteristic of host–guest inclusion complexation were observed in the FTIR spectra, suggesting that β-cyclodextrin (BCD) acts primarily as a stabilizing and dispersing agent rather than forming a defined inclusion complex with ATH. The broad O–H and C–O stretching bands of BCD indicate the presence of weak hydrogen bonding and van der Waals interactions with ATH and the surrounding polymer chains. Such non-covalent interactions help prevent ATH aggregation, enhance its uniform distribution, and improve its compatibility within the hydrophilic–hydrophobic PVP–EC matrix. This configuration therefore represents a multi-component physical mixture, which simplifies fabrication while ensuring homogeneous incorporation of ATH during electrospinning.

The role of BCD in this system is to provide a microenvironment that partially shields ATH from degradation and maintains its optical responsiveness without requiring the formation of a host–guest supramolecular complex. Similar stabilizing effects of BCD have been reported in other polymer-based systems where physical interaction, rather than inclusion, improved dispersibility and photostability of natural dyes and bioactive molecules.^88^

To provide a clear summary, Table 2 lists the major FTIR peaks observed for PVP, EC, BCD, and ATH. PVP contains carbonyl groups, while EC has free hydroxyl groups, both of which are capable of forming proton donor–acceptor interactions. Due to the presence of carbonyl and hydroxyl groups, inter- and intramolecular hydrogen bonding may play a critical role in maintaining the structure of the ATH-loaded PVP:EC:BCD nanofiber systems.^89^

**Table 2:** Summary of the major FTIR peaks of PVP, EC, BCD, and ATH

Compound	Wavenumber (cm^−1^)	Functional group	Vibration type	Reference
PVP	1651	C=O	Stretching	65
	1284	C–N	Stretching	78
	3434	O–H	Symmetric stretching	79
	2950	C–H	Symmetric stretching	79
	1017	C–H_2_	Rocking vibration	90
	1225	C–H_2_	Twisting vibration	90
EC	1052	C–O–C	Stretching	65
	1375	C–H	Bending	65
	2972, 2872	C–H	Stretching	65
	3485	O–H	Stretching	91
BCD	3431	O–H	Vibration (hydroxyl group)	80
	2927	C–H	Stretching	80
	1155, 1083, 1036	C–O	Coupled vibrations	80
		C–O–C		
		C–C–O		
		C–C–C		
	1020	C–C	Stretching	92
ATH	1017	C–O(H)	Stretching	81
	2500–3434	O–H	Stretching	82
	1627–1693	C=O	Stretching	82
	2928	C–H	Asymmetric stretching	82

### 3.3 XRD analysis

**Fig. 3:**
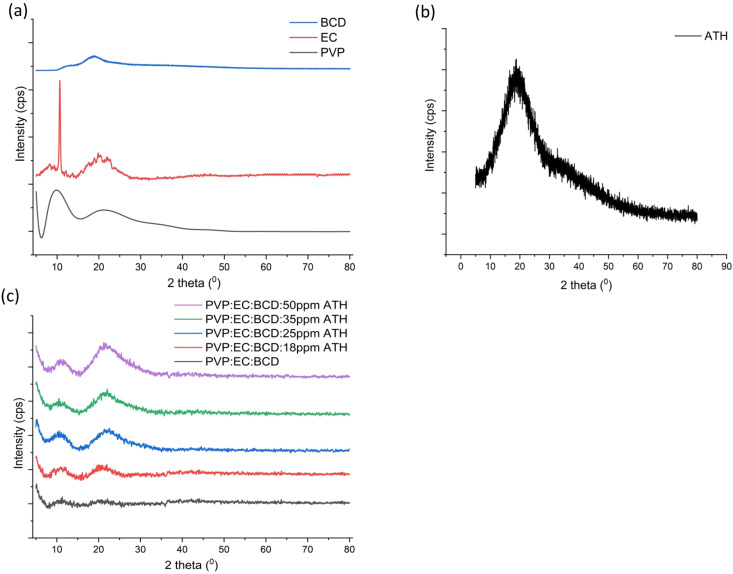
(a) XRD pattern of neat polymers. (b) XRD pattern of neat ATH. (c) XRD patterns of the PVP:EC:BCD nanofiber mat and 18 ppm, 25 ppm, 35 ppm, and 50 ppm ATH incorporating PVP:EC:BCD nanofiber mats.

XRD analysis was employed to investigate the crystallinity and structural organization of the neat polymers, electrospun nanofiber mats and ATH. The XRD patterns obtained are depicted in Fig. 3. The PVP neat polymer (see Fig. 3(a)) exhibited two broad peaks at 10° and 20.5°, indicating its amorphous nature.^79^ The typical XRD pattern of EC further exhibits noticeable Bragg reflections between ≈11° and a broad peak at 20° (2*θ*), confirming the presence of short-range molecular ordering typical of semi-crystalline EC.^93^ Similar patterns have also been reported by Trivedi *et al.* (2015) and Qosim *et al.* (2024), supporting that the peaks observed in Fig. 3(a) arise from the intrinsic semi-crystalline nature of EC.^94,95^ The EC neat polymer, in this study (Fig. 3(a)) displayed a characteristic peak at 10.67° and a broad peak at 19.83°, indicating the semi-crystalline nature of the polymer.^91^ According to the literature, ATH has an amorphous nature and therefore gives a broad peak around 20° as shown in Fig. 3(b).^74^ The PVP:EC:BCD electrospun nanocomposite exhibits an amorphous nature. Similar studies suggested that products obtained by electrospinning of PVP and EC usually exhibit an amorphous nature due to rapid solvent evaporation during electrospinning and the short travelling time before depositing the polymers and ingredients.^65,74,78,79^ This indicates that polymers and incorporated molecules in the nanocomposite do not have enough time to arrange in a regular crystalline structure during the electrospinning.

The XRD patterns further confirm that the obtained nanofiber mats represent a multi-component physical mixture rather than a crystalline inclusion complex between ATH and BCD. The absence of additional diffraction peaks that would indicate a new crystalline phase confirms that BCD remains molecularly dispersed within the PVP–EC network.^95^ This homogeneous dispersion supports efficient stabilization of ATH without altering its intrinsic amorphous characteristics. Such amorphous, physically mixed systems have been reported to enhance the optical responsiveness and environmental stability of encapsulated molecules, particularly natural pigments, while avoiding the need for complex synthesis steps associated with pre-formed inclusion complexes.^96^

However, in this study, a slight increase in crystallinity was observed at around 20° (Fig. 3(c)) after the incorporation of ATH into the polymer matrices, which is similar to the peak appearing in the neat ATH XRD diffractogram shown in Fig. 3(b). This may result from the plasticizing effect of ATH, which refers to its ability to weaken intermolecular electrostatic interactions.^97^ Similar studies, such as that of Xu *et al.* (2025), reported that the crystallinity of their ATH-incorporating nanofibers increased after the ATH concentration exceeded 6%.^97^ Additionally, ATH molecules may engage in intramolecular interactions, such as hydrogen bonding with these polymers. As a result, increasing the concentration of ATH could promote the formation of a more ordered structure rather than maintaining the random orientation of the polymer chains. Therefore, it can be deduced that the increment in ATH concentration led to a slight increase in the crystallinity of the PVP:EC:BCD nanofibers and the electrospun nanofibers still maintain sufficient flexibility and amorphous characteristics necessary for their intended applications.

### 3.4 Thermogravimetric analysis (TGA)

**Fig. 4:**
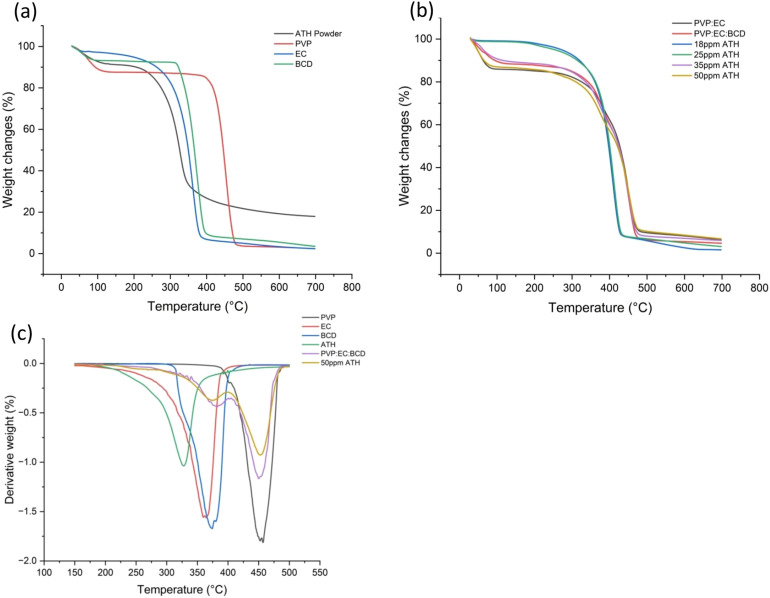
TGA analysis of electrospun nanofibers. (a) TGA analysis of ATH powder and neat polymers. (b) TGA analysis of neat polymer blends and polymer blends incorporating different ATH concentrations (18 ppm, 25 ppm, 35 ppm, and 50 ppm). (c) DTG analysis of neat polymers, ATH, PVP:EC:BCD, and 50 ppm ATH incorporating PVP:EC:BCD.

The thermal degradation patterns of electrospun nanofibers are depicted in Fig. 4(a) and (b). Both Fig. 4(a) and (b) show the initial weight loss of all samples occurred between 50 °C and 100 °C due to the removal of physically absorbed water.^98^ The second weight loss region between 150 °C and 600 °C represents decomposition of the precursors. Neat PVP undergoes decomposition in the temperature range of 300 °C to 600 °C, while other neat polymers exhibit thermal decomposition between 200 °C and 400 °C.^85–87^ After incorporating different ATH concentrations into the PVP–EC–BCD polymer blend, the decomposition temperature has shifted to a higher value. For 18 ppm, it was at 227 °C with a total weight loss of 30%, while it was 290 °C, 345 °C, and 350 °C for 25 ppm, 35 ppm, and 50 ppm, respectively, accounting for weight loss percentages of 16%, 22% and 23%.

These results indicate a clear enhancement in thermal stability with increasing ATH concentration. Further insights from the differential thermogravimetric (DTG) analysis presented in Fig. 4(c) support this observation. The DTG curves show peak decomposition temperatures as follows: PVP – 457 °C, EC – 366 °C, BCD – 374 °C, ATH – 326 °C, PVP:EC:BCD – 450 °C, and PVP:EC:BCD with 50 ppm ATH – 454 °C. These values confirm that the 50 ppm ATH-loaded nanofiber mat exhibits a higher decomposition temperature than most neat polymers (except PVP) and slightly improved thermal stability compared to the PVP:EC:BCD system without ATH.^99^ Our results suggest a high thermal stability for ATH-incorporating PVP:EC:BCD nanofibers.

### 3.5 Zebrafish toxicity analysis (FET 236 acute toxicity assessment)

**Fig. 5:**
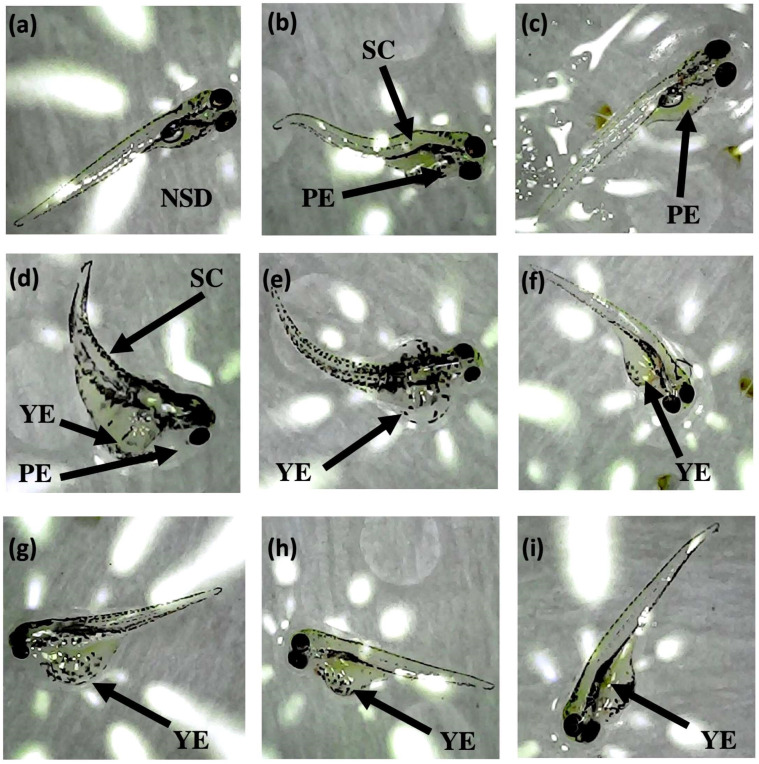
Deformities developed after exposing zebrafish embryos to different ATH concentrations incorporated in PVP–EC–BCD mats. (a) No structural deformities (NSD) in control embryos. (b) and (c) Developmental abnormalities in zebrafish embryos exposed to a PVP:EC:BCD neat polymer mat showing pericardial edema (PE) and spinal scoliosis. (d) 18 ppm ATH incorporating polymer mat showing pericardial edema (PE), yolk sac edema (YSE), and spinal scoliosis (SC). (e)–(h) Yolk sac edema (YSE) developed at 25 ppm of ATH. (h) and (i) Yolk sac edema (YSE) developed at 35 ppm and 50 ppm ATH concentrations.

The acute toxicity studies of the developed pH-sensitive electrospun nanofiber mats were conducted using zebrafish embryos. The acute toxicity analysis was conducted to verify that the pH-sensitive nanofiber mat does not pose any toxicity risks when applied to sensitive areas, such as the eye. Experiments were performed according to the guidelines of the Organisation for Economic Co-operation and Development (OECD) 2013 following FET236 acute toxicity assessment guidelines. All zebrafish experiments were conducted under the standard mini-zebrafish rearing system at the Centre for Advanced Materials and Devices (CAMD), Department of Chemistry, University of Colombo. All experimental procedures complied with the Guidelines for the Care and Use of Laboratory Animals and were approved by the Animal Ethics Committee of the Institute of Biology (IOB), Sri Lanka, under ethical application number ERC IOBSL 19907 2019. This study demonstrates various types of embryonic developmental toxicity anomalies in fish exposed to different concentrations of ATH incorporated into the developed pH-sensitive material, as illustrated in Fig. 5. This figure provides a visual depiction of the developmental deformities observed in zebrafish embryos following exposure to various concentrations of ATH-incorporating PVP–EC–BCD mats. No structural deformities were observed in the control group (Fig. 5(a)); however, in embryos exposed to the neat polymer mat without ATH (Fig. 5(b) and (c)), pericardial edema (PE) and spinal scoliosis (SC) were noted, indicating the potential toxicity of the polymer itself. At an ATH concentration of 18 ppm (Fig. 5(d)), the embryos exhibited pericardial edema, yolk sac edema (YSE), and spinal scoliosis. As the concentration of ATH increased to 25 ppm (Fig. 5(e)–(h)), yolk sac edema became the predominant deformity, with increasing severity at 35 ppm and 50 ppm (Fig. 5(i)). These findings suggest that while the neat polymer may cause deformities, adding ATH introduces specific anomalies, particularly at higher concentrations.

**Fig. 6:**
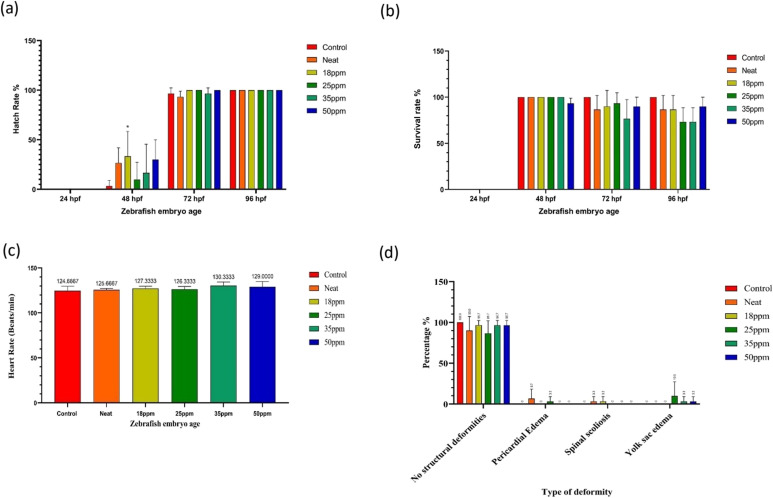
Zebrafish toxicity assay statistical analysis of the developed pH-sensitive material: (a) hatching rate analysis, (b) survival rate analysis, (c) heart rate analysis and (d) deformity analysis.

The statistical analysis of the zebrafish toxicity assay is presented in Fig. 6. Despite the occurrence of deformities, especially yolk sac edema, was prominently observed with increasing ATH concentrations. However, the frequency and severity did not correlate with a statistically significant increase in overall teratogenic effects, which is the ability of a substance to cause congenital deformities. Compared with the control, there is no significant difference in the hatching rate of embryos exposed to ATH concentrations of 18 ppm, 25 ppm, 35 ppm, and 50 ppm, and the neat polymer (PVP:EC:BCD) fiber mats (see Fig. 6(a)). Similarly, the survival rate of embryos did not show any significant differences across all tested ATH concentrations at any time interval. This suggests that while hatching rates remained unaffected at all tested ATH concentrations, overall embryo viability also remained largely unaffected, as shown in Fig. 6(b). According to the heart rate analysis depicted in Fig. 6(c), there were no significant differences across all ATH concentrations compared to the control. This suggests that ATH exposure did not considerably affect zebrafish cardiac function within the parameters tested. According to Fig. 6(d), deformities observed in the exposed embryos included spinal scoliosis, pericardial edema, and yolk sac edema, with no notable variation between samples with different ATH concentrations and the neat polymer samples. Yolk sac edema was observable more frequently in embryos exposed to a 25 ppm ATH concentration compared to other anomalies, but this difference was not statistically significant compared to the controls at any time interval.

Our results suggest that ATH exposure within the concentration range of 18 ppm to 50 ppm did not significantly affect hatching rates or survival across all tested concentrations, nor did it significantly impact the heart rate. The deformities observed, including spinal scoliosis, pericardial edema, and yolk sac edema, were consistent across all concentrations and the neat polymer, with no statistically significant differences compared to the control. Accordingly, ATH had not imposed any physiological alterations in zebrafish larvae within this dose range. These findings suggest that all tested ATH concentrations (18, 25, 35, and 50 ppm) had no substantial teratogenic risk to zebrafish embryos, exhibiting biocompatibility.

### 3.6 Color variation analysis

**Fig. 7:**
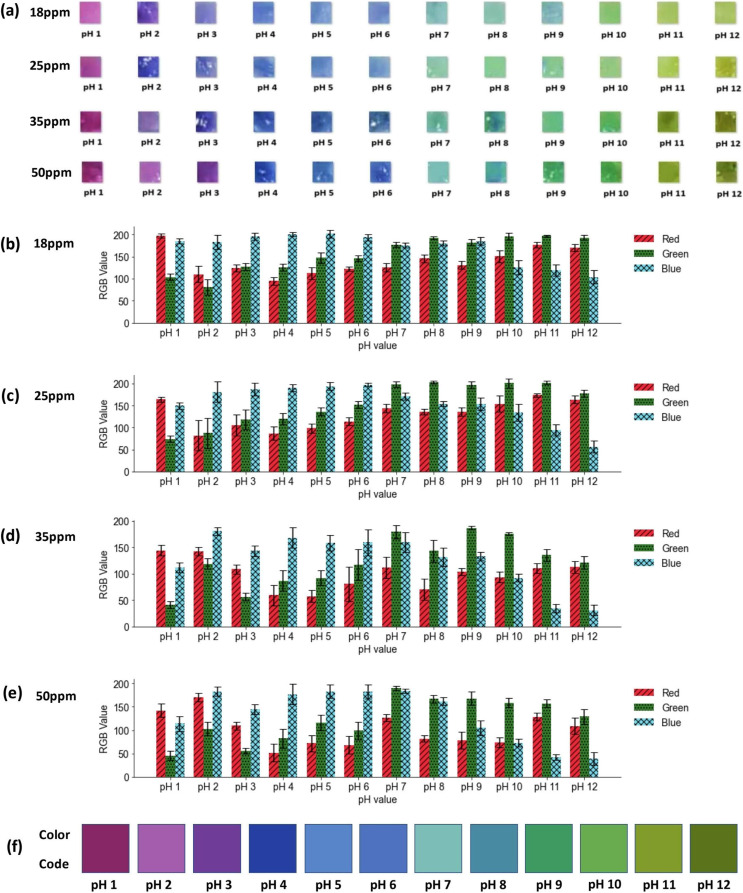
Color variation analysis of ATH-incorporating electrospun nanofiber mats across a pH range of 1–12. (a) Visual color responses of nanofiber mats containing ATH concentrations of 18, 25, 35, and 50 ppm. (b–e) Corresponding RGB analysis graphs for each concentration. (f) Developed reference color code.

The color variation analysis aimed to evaluate the sensitivity and responsiveness of ATH-incorporating electrospun nanofiber mats to pH fluctuations, an essential characteristic for their potential application as ocular pH sensors. By quantifying color responses across a broad pH range (1–12), the analysis provided valuable insights into the ability of nanomats to detect and visually represent pH changes. This functionality is critical for the rapid identification and treatment of ocular conditions such as chemical injuries. Fig. 7(a) illustrates the visual color-changing performance of pH-sensitive nanofiber mats containing ATH at concentrations of 18, 25, 35, and 50 ppm. Fig. 7(b)–(e) present the corresponding RGB analyses for each concentration, demonstrating the variation in the color intensity across the pH range. The results reveal distinct color change patterns that depend on both pH level and ATH concentration. At 18 ppm, Fig. 7(a) shows moderate visual fluctuations, while Fig. 7(b) confirms these changes through RGB analysis. However, color variations between pH 2 and 6 are less distinguishable to the naked eye, indicating reduced sensitivity to acidic environments. This is further supported by the prevalence of the blue bars in the RGB graph. While pH 6 and 7 exhibit noticeable color differences (confirmed by varying RGB values), pH 7 to 9 show minimal variation, aligning with limited visual distinction. A significant difference is observed between pH 9 and 10, whereas pH 10 to 12 show reduced color differentiation, as reflected by similar RGB bar heights.

In the 25 ppm series, Fig. 7(c) and the corresponding visual data in Fig. 7(a) indicate a discernible colour response resulting from the increased ATH concentration. Nevertheless, the pH 2–6 range still presents limited visual distinguishability, as supported by similar RGB bar heights. A visual distinction was observed between pH 6 and 7, marked by a shift in prominent RGB components from blue at pH 6 to green at pH 7, highlighting the transition. However, the color responses between pH 7 and 10 appear similar in both visual and RGB data. Distinct differences were seen between pH 10 and 11, as indicated by increased red and green values, while the distinction between pH 11 and 12 was supported by a decrease in the blue bar height. At 35 ppm, some of the color responses became distinguishable across the range, as shown in Fig. 7(a). pH 2 shows a clear change, while pH 4 to 6 remains less distinct, confirmed by the similar RGB bar heights in Fig. 7(d). Notably, pH 7 to 9 revealed recognizable visual and RGB variations, while pH 9 and 10 showed minimal change. A noticeable color difference was observed between pH 10 and 11. However, the variation becomes less distinct between pH 11 and 12. The 50 ppm series demonstrates effective color responsiveness across both acidic and basic pH levels. As shown in Fig. 7(a) and further validated by the RGB analysis in Fig. 7(e), distinct variations in RGB quantifications were well correlated with each pH value. This confirmed enhanced color fluctuation at every pH level, supporting the 50 ppm ATH concentration as optimal for broad-range pH detection. The results indicate that this concentration provides enhanced sensitivity, making it especially effective for detecting changes in ocular pH during chemical exposure. In addition, a standardized color reference code was developed based on the performance of the 50 ppm ATH-incorporating nanofiber mats across the tested pH values. This reference, presented in Fig. 7(f), serves as a visual guide to support the interpretation of pH levels in real-world scenarios. Such a reference can be used in practical applications to improve the speed and accuracy of ocular pH assessment in clinical or emergency settings. Considering the need for precise and immediate pH detection in cases of accidental ocular chemical spillages, the 50 ppm ATH-incorporating nanofiber mats stood out due to their clear, observable color responses across the entire pH spectrum. Their robust performance under both acidic and alkaline conditions makes them ideal for such critical applications.

### 3.7 Shelf-life evaluation

The shelf-life evaluation of the pH-sensitive electrospun nanofiber mats examined in this study revealed that storage conditions have a significant impact on the long-term stability and pH responsiveness of the materials. Over the three month evaluation period, the mats were tested at two week intervals under different storage conditions, such as dark, light, and refrigeration. The main objective of this study was to identify the optimal storage condition that maintains the pH-sensitive properties of the mats over time while minimizing degradation. The storage environments were characterized as follows: refrigeration at approximately 4 ± 1 °C, light exposure under ambient room conditions at 28 ± 2 °C with relative humidity (RH) of 70–80%, and dark storage in a closed container at room temperature (28 ± 2 °C, RH 65–75%) to minimize light penetration.

According to the analysis (Fig. S3 in the SI section), it was found that mats stored under refrigeration showed the most stable performance in terms of pH responsiveness. Throughout the three months, the pH-sensitive color changes of these mats remained consistent across all tested pH levels (3, 5, 7, 9, and 11). Nevertheless, the mats stored under dark and light conditions exhibited a gradual decline in their pH sensitivity, with noticeable degradation starting as early as the fourth week in light-exposed samples. This suggests that prolonged exposure to light may accelerate the breakdown of ATH within the fibers, leading to reduced pH sensitivity. Similarly, the mats stored under dark conditions maintained their pH responsiveness better than those exposed to light but still showed minor degradation in color intensity after six weeks.

One of the significant findings of this study was the pH response time. The mats stored in refrigeration consistently demonstrated a rapid response to pH changes, with a reaction time of less than 5 seconds, even after three months of storage. In contrast, mats stored under light conditions exhibited a slower response time, particularly after eight weeks, where the color change time increased to around 10–15 seconds, indicating a loss of reactivity. Mats stored under dark conditions showed a moderate response time, with slight increases in reaction time (∼6–8 s) observed after six weeks of storage. The faster pH response time observed in refrigerated mats is likely due to the preservation of ATH's structural integrity, which is sensitive to environmental conditions such as temperature and light.^89,100^ The findings suggest that refrigeration helps preserve both the color intensity and the pH-sensitive functionality of the nanofiber mats, making it the most suitable storage condition for long-term applications. These results further highlight the importance of controlling storage conditions to ensure the continued efficacy of pH-sensitive nanomaterials in practical applications, such as medical diagnostics or environmental monitoring.

## 4 Conclusions

This study developed a novel ATH-incorporating PVP–EC–BCD electrospun nanofiber mat as a rapid and biocompatible pH sensor for ocular applications. The sensor exhibited uniform nanofiber morphology with mean diameters ranging from 201.97 ± 18.74 nm to 390.79 ± 13.31 nm and demonstrated successful ATH incorporation, as verified by FTIR and XRD analyses. Thermal and biocompatibility evaluations confirmed improved stability and non-toxicity within the tested ATH concentration range (18–50 ppm). The 50 ppm ATH formulation provided the most distinct and reversible color transitions over a full pH range (1–12), with rapid response and excellent shelf stability under refrigerated conditions.

Compared with conventional ocular pH detection methods such as litmus papers, nitrazine strips, bromothymol blue assays, and the glass probe method, the proposed nanofiber-based system offers enhanced sensitivity, rapid visualization, and practical adaptability for clinical and emergency settings. The integration of natural pH-responsive ATH pigments into a biocompatible nanofiber matrix presents a sustainable and minimally invasive alternative to existing technologies. Future work will focus on enhancing sensor stability under physiological conditions and expanding its applicability to other biomedical sensing applications.

## Author contributions

Benuwan Sandaruwan: conceptualization, methodology, investigation, data curation, formal analysis, writing – original draft preparation, writing – review & editing. Danushika C. Manatunga: conceptualization, supervision, writing – review & editing, funding acquisition. Rohan S. Dassanayake and Ruchire Eranga Wijesinghe: conceptualization, supervision, writing – review & editing. Renuka Liyanage, Pabakara Costha, H. M. L. P. B. Herath, K. M. Nalin de Silva, Rohini M. de Silva, and Suranga M. Rajapaksha: conceptualization, supervision, visualization, validation. Udaya Wijenayake: conceptualization, supervision, funding acquisition. All authors have approved the final version of the manuscript.

## Conflicts of interest

The authors declare that there is no conflict of interest.

## Supplementary Material

NA-008-D5NA00819K-s001

NA-008-D5NA00819K-s002

NA-008-D5NA00819K-s003

NA-008-D5NA00819K-s004

## Data Availability

The authors declare that all data supporting the findings of this study are available within the article and its supplementary information (SI). Supplementary information: includes chemical structure illustrations of PVP, EC, BCD, and ATH (Fig. S1), enlarged FTIR spectra in the 700–1500 cm^−1^ region (Fig. S2), and shelf-life evaluation data of pH-sensitive electrospun nanofiber mats aganist time (Fig. S3). See DOI: https://doi.org/10.1039/d5na00819k.
