# Supplementary figures and images for: Anthocyanin (ATH)-incorporating polyvinylpyrrolidone-ethyl cellulose-(2-hydroxypropyl)-β-cyclodextrin (PVP–EC–BCD) nanofiber-based pH sensor for ocular pH detection during accidental chemical spills
Source: Nanoscale Adv. 2025 Nov 26;8(3):945–60. doi: 10.1039/d5na00819k (PMC12713680; doi:10.1039/d5na00819k)

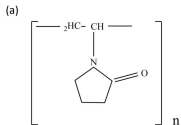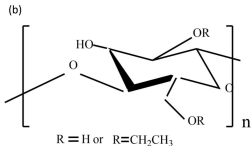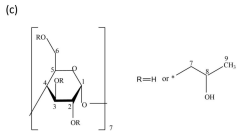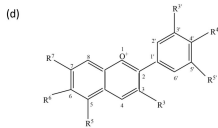

Supplement: NA-008-D5NA00819K-s001 [file NA-008-D5NA00819K-s001.pdf]

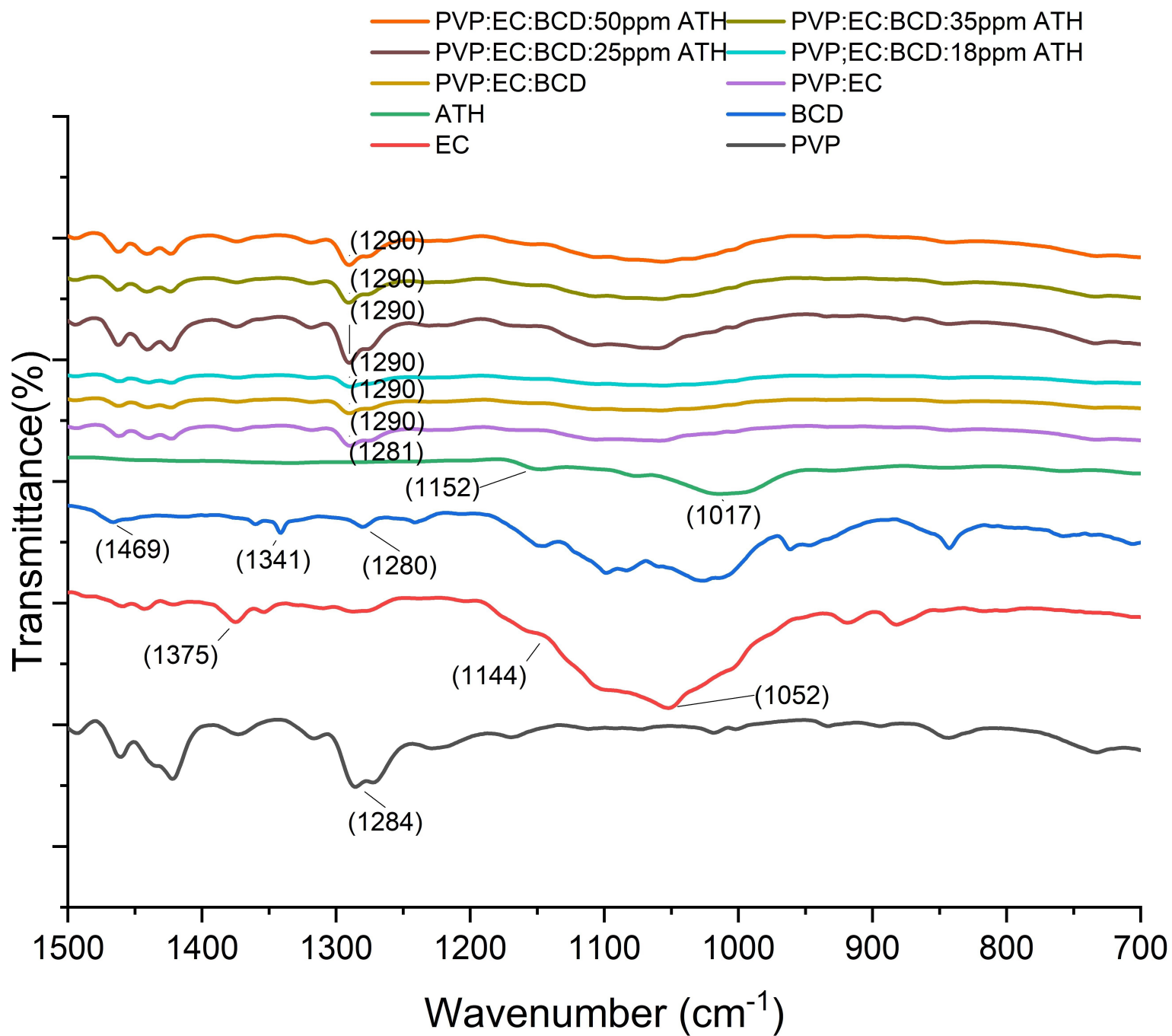

Supplement: NA-008-D5NA00819K-s002 [file NA-008-D5NA00819K-s002.pdf]

Step 1

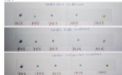

After 4 weeks

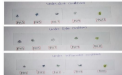

After 8 weeks

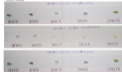

After 12 weeks

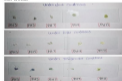

After 2 weeks

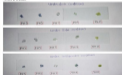

After 6 weeks

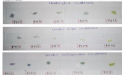

After 10 weeks

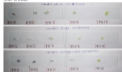

Supplement: NA-008-D5NA00819K-s003 [file NA-008-D5NA00819K-s003.pdf]
