# Supplementary material for: Anthocyanin (ATH)-incorporating polyvinylpyrrolidone-ethyl cellulose-(2-hydroxypropyl)-β-cyclodextrin (PVP–EC–BCD) nanofiber-based pH sensor for ocular pH detection during accidental chemical spills
Source: Nanoscale Adv. 2025 Nov 26;8(3):945–60. doi: 10.1039/d5na00819k (PMC12713680; doi:10.1039/d5na00819k)
Supplement: NA-008-D5NA00819K-s004 [file NA-008-D5NA00819K-s004.pdf]

Supplementary Information (SI) for Nanoscale Advances.
This journal is © The Royal Society of Chemistry 2025

Anthocyanin (ATH)-incorporated polyvinylpyrrolidone-ethyl cellulose-(2-Hydroxypropyl)-β-cyclodextrin (PVP-EC-BCD) nanofibers-based pH sensor for ocular pH detection during accidental chemical spills

Supplementary Material

Benuwan Sandaruwan [a], Renuka Liyanage [b], Pabakara Costha [c], Rohan S. Dassanayake [a], Ruchire Eranga Wijesinghe [d,e], H. M. L. P.B. Herath [f], K.M. Nalin de Silva [f], Rohini M. de Silva [f,g], Suranga M. Rajapaksha [b], Udaya Wijenayake [h] and Danushika C. Manatunga [a,f]*

[a] Department of Biosystems Technology, Faculty of Technology, University of Sri Jayewardenepura, Pitipana North, Homagama 10200, Sri Lanka.

[b] Department of Materials and Mechanical Technology, Faculty of Technology, University of Sri Jayewardenepura, Pitipana North, Homagama 10200, Sri Lanka.

[c] National Eye Hospital of Sri Lanka, Colombo, 01000, Sri Lanka.

[d] Center for Excellence in Informatics, Electronics & Transmission (CIET), Sri Lanka Institute of Information Technology, Malabe 10115, Sri Lanka.

[e] Department of Electrical and Electronic Engineering, Faculty of Engineering, Sri Lanka Institute of Information Technology, Malabe, 10115, Sri Lanka.

[f] Center for Advanced Materials and Devices (CAMD), Department of Chemistry, University of Colombo, PO Box 1490, University of Colombo, Colombo 00300, Sri Lanka.

[g] Department of Life Sciences, Faculty of Science, NSBM Green University, Homagama, 10200, Sri Lanka

[h] Department of Computer Engineering, Faculty of Engineering, University of Sri Jayewardenepura, Nugegoda 10250, Sri Lanka.

Corresponding author E-mail: danushi@sjp.ac.lk

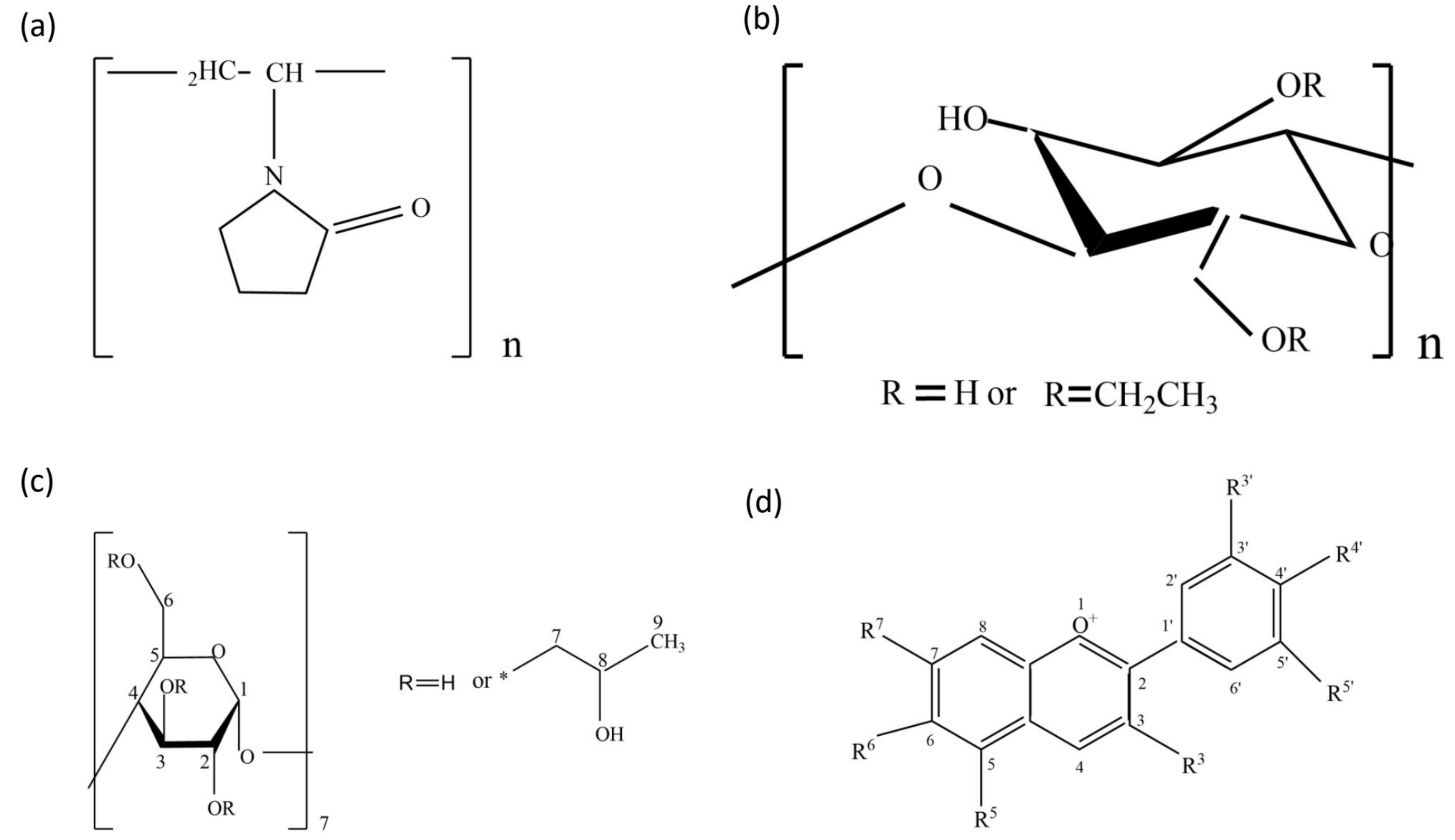

Figure S1. Chemical structures of (a) PVP (b) EC (c) BCD (d) ATH

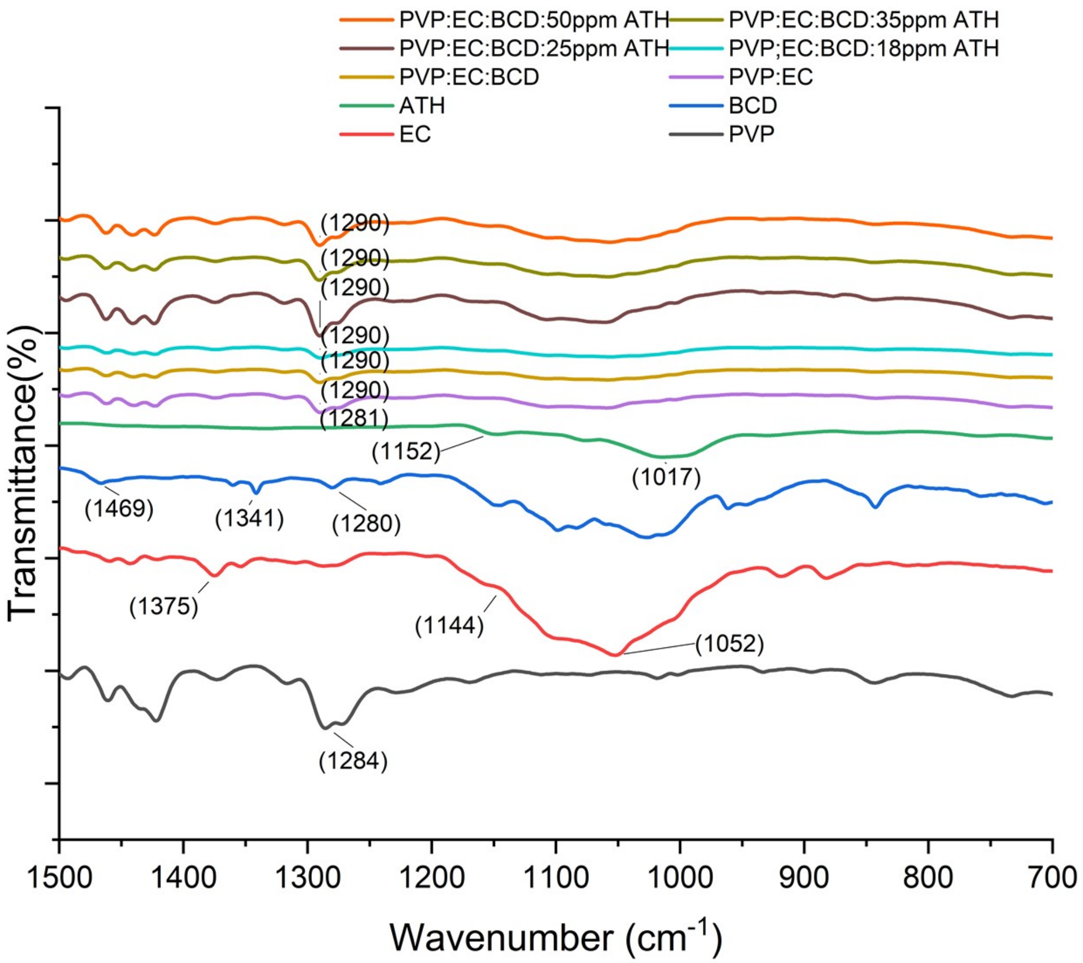

Figure S2. Enlarged FTIR spectra of the samples in the 700-1500 cm-1 wavelength range.

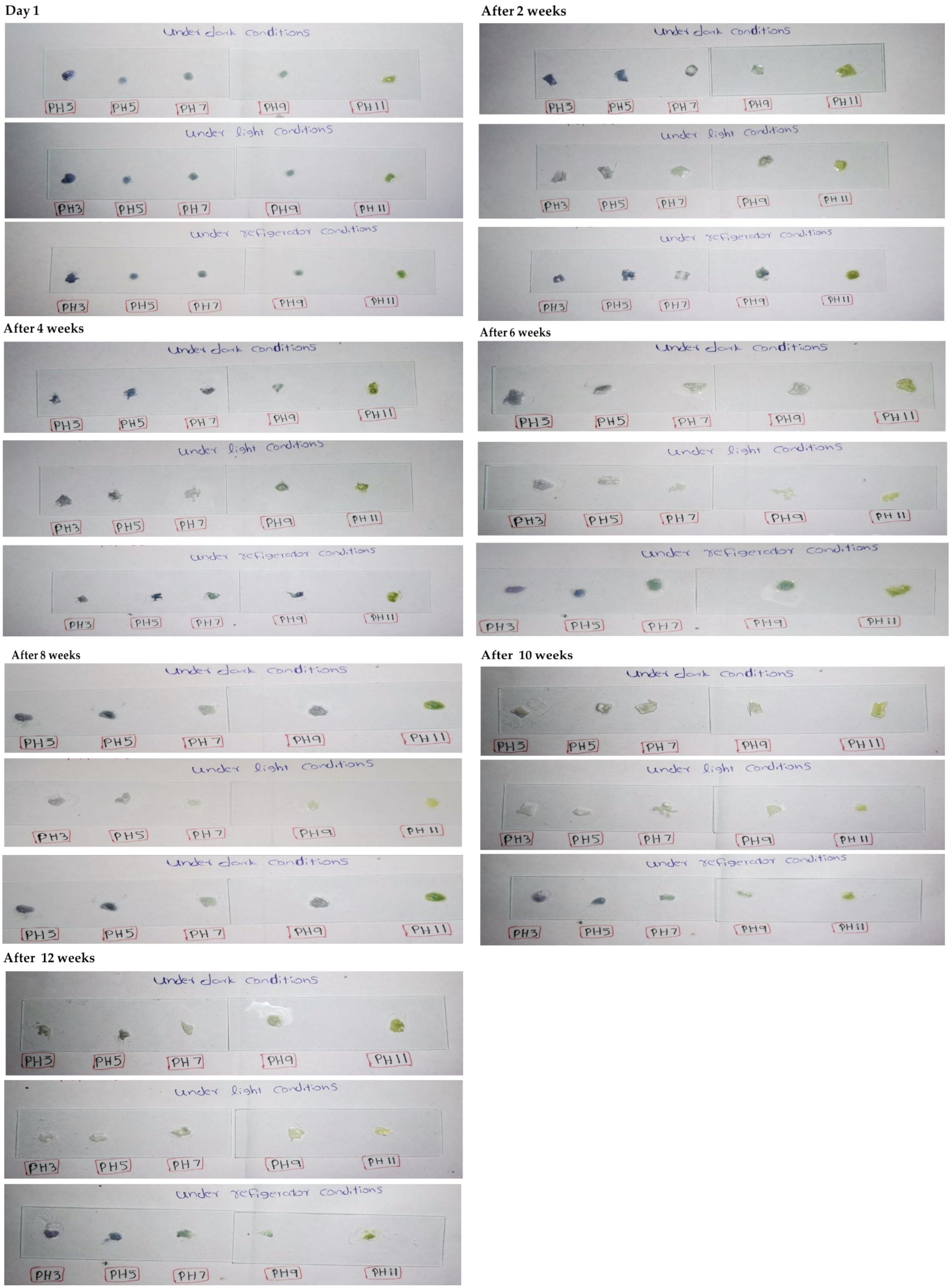

Figure S3. Shelf life evaluation of pH-sensitive electrospun nanofiber mats over time points of day 1, 2 weeks, 4 weeks, 6 weeks, 8 weeks, 10 weeks, and 12 weeks.
